# Supplementary material for: An ultrapotent RBD-targeted biparatopic nanobody neutralizes broad SARS-CoV-2 variants
Source: Signal Transduct Target Ther. 2022 Feb 9;7:44. doi: 10.1038/s41392-022-00912-4 (PMC8828845; doi:10.1038/s41392-022-00912-4)
Supplement: Supplementary file 1 — Supplementary Materials [file 41392_2022_912_MOESM1_ESM.docx]

Supplementary Materials for

An ultrapotent RBD-targeted biparatopic nanobody neutralizes broad SARS-CoV-2 variants

Xiaojing Chi^1,#^, Xinhui Zhang^1,#^, Shengnan Pan^1,#^, Yanying Yu^2,#^, Yujin Shi^1,#^, Tianli Lin^1^, Huarui Duan^1^, Xiuying Liu^1^, Wenfang Chen^1^, Xuehua Yang^1^, Lan Chen^1^, Xiaoqian Dong^1^, Lili Ren^1^, Qiang Ding^2,*^, Jianwei Wang^1,*^,Wei Yang^1,3,*^

Correspondence to: wyang@ipb.pumc.edu.cn

**This PDF file includes:**

Materials and Methods

Figures. S1 to S4

Tables S1 to S3

Materials and Methods

**Cells and reagents.**

The HEK293T (human kidney epithelial) cells were obtained from China Infrastructure of Cell Line Resource (Beijing, China). The human hepatoma cell line Huh7 was obtained from Apath, Inc (Brooklyn, NY, USA) with permission from Dr. Charles Rice (Rockefeller University). The Expi293F cells were purchased from ThermoFisher (Waltham, MA, USA). The cells were maintained in Dulbecco's modified Eagle's medium (ThermoFisher) supplemented with 2-10% fetal bovine serum (FBS, ThermoFisher), non-essential amino acid, penicillin and streptomycin. Recombinant RBD and ACE2 proteins were purchased from Sino Biological (Beijing, China). HRP/anti-CM13 monoclonal conjugate was from GE Healthcare (Boston, MA, USA).

**Expression and purification of nanobodies.**

Full-length sequences of selected nanobodies were PCR amplified and cloned into the NcoI/XhoI sites of the pET28b (Novagen, Sacramento, CA, USA) and transformed into BL21(DE3) chemically competent E. coli. The expression of recombinant nanobodies was induced by adding IPTG to a final concentration of 0.3 mM after culture had reached OD600=0.5-0.6 and grown over night at 25°C. The nanobodies were fused with a His-tag at C-terminus and purified over Ni Sepharose 6 Fast Flow (GE Healthcare) and eluted with 400 mM imidazole. Affinity purified sdAbs were dialyzed against PBS to eliminate imidazole.

**Construction of bivalent and Fc-fused nanobodies**

To improve the neutralization activity of Nbs, we constructed dimeric nanobodies with various combinations and a (GGGGS)_5_ linker was introduced between the two monomers. The recombinant bivalent nanobodies were produced in E *coli*. and a His-tag was designed to facilitate purification. In addition, the sequence of dimeric Nb1-Nb2 was cloned into a mammalian expression vector under the control of hEF1-HTLV promotor and fused with N-terminal interleukin-2 signal peptide and C-terminal Fc region, comprising the CH2 and CH3 domains of human IgG1 heavy chain and the hinge region. Maxiprepped plasmids were transiently transfected into Expi293F cells (Thermofisher) and the cells were further cultured in suspension for 2-3 days before harvesting antibody-containing supernatant. Fc-fused nanobody was prepared with prepacked HiTrap® Protein A HP column (GE Healthcare). The produced Fc-fusion protein was analyzed by SDS-PAGE using standard protocols for dimerization, yield and purity measurement.

**Biolayer interferometry (BLI)** **measurement**

Antibody affinity analysis was conducted by ForteBio Octet RRD 96 system. The VOC/VOI derived RBD recombinant proteins (Sino Biological, Cat: 40592- V08H / V08H82 / V08H85 / V08H86 / V08H88 / V08H90) were diluted in 10 mM Acetate pH 5.5 buffer at a density of 10μg/ml. The Amine Reactive 2^nd^ Generation biosensors surface was activated with EDC and NHS, then immobilized the RBD proteins for 5 min. Following 10 s of baseline in kinetic buffer (KB: 1× PBS, 0.01% BSA, and 0.02% Tween-20), the loaded biosensors were dipped into serially diluted (3.125–50 nM) nanobodies for 120 s to record association kinetics. The sensors were then dipped into kinetic buffer for 180 s to record dissociation kinetics. Kinetic buffer without antibody was set to correct the background. The Octet Data Acquisition 9.0 was used to collect affinity data. The mean Kon, Koff, and apparent KD values were calculated using an equation globally fitted to a 1:1 binding kinetic model and using the global fitting method.

**Epitope competition-binding Study.**

For the ACE2 competition assay, the RBD-immobilized biosensors were then dipped into the wells containing 100 nM of ACE2 for a 360-s association period. The sensors were then transferred to wells containing 100nM ACE2 or 100nM ACE2 +100nM Nb samples and incubated for 400s. For all BLI assays, data analysis was performed using Octet data analysis software version 11.0 (Pall FortéBio).

**Stability tests**

Antibody samples diluted in PBS (1 mg/mL) were filtrated and sealed in a 1.5 mL Eppendorf tube and stored at 37°C for 3 or 6 days. At the end of the storage period, samples were centrifuged (10,000× g) for 10 min and neutralization activities were evaluated using pseudovirus.

**Circular Dichroism measurements**

CD spectral data of the protein solution was obtained using the Spectra Measurement program on a Jasco J-815 CD spectrometer equipped with a 1.0 mm path length unit. HBS solution with 20 mmol/L concentration was mixed with Nano antibodies separately so that the final concentration was 15 μmol/L. The wavelength range from 200 nm to 250 nm was scanned and far-ultraviolet spectrum data was collected. SpectraManger software was used to process the collected data to obtain the content of the circular chromatogram of each system. Temperature regulation was carried out using the Variable Temperature Measurement program. A data pitch of 0.1 nm and bandwidth of 1 nm was used. Heat-induced unfolding was recorded at 208 or 218 nm, and a heating rate of 0.5°C/min was used.

**Antibody-escape sites visualization**

The RBD and ACE2 binding crystal structure (PDB: 6M0J) is represented by a surface pattern. The antibody-escape amino acids on RBD are colored at each site. Different degrees of escape are indicated in different colors. Red represents complete escape, pink represents moderate escape, and light pink represents weak escape. Interactive visualizations of the escape maps and their projection onto the ACE2-bound were created using dms-view (https://dms-view.github.io/docs/).

**Statistics and reproducibility.**

Data were analyzed using GraphPad Prism 6.01 (GraphPad Software, San Diego, CA, USA). The values shown in the graphs are presented as means ± SD. One representative result from at least two independent experiments was shown. Antibody neutralization experiments usually use three to four duplicated wells for each treatment. The infectivity data were first inversed to neutralization activity. Each neutralization data set was normalized by the background control (no virus) to define the real value for 100% neutralization. After transformation to neutralization, the lowest concentration point of antibody treatment was set to 0% neutralization. Then, a 4-parameters neutralization nonlinear regression model was fitted to report IC_50_ values. All experiments were performed independently at least twice and similar results were obtained. One representative data of one experiment were shown.


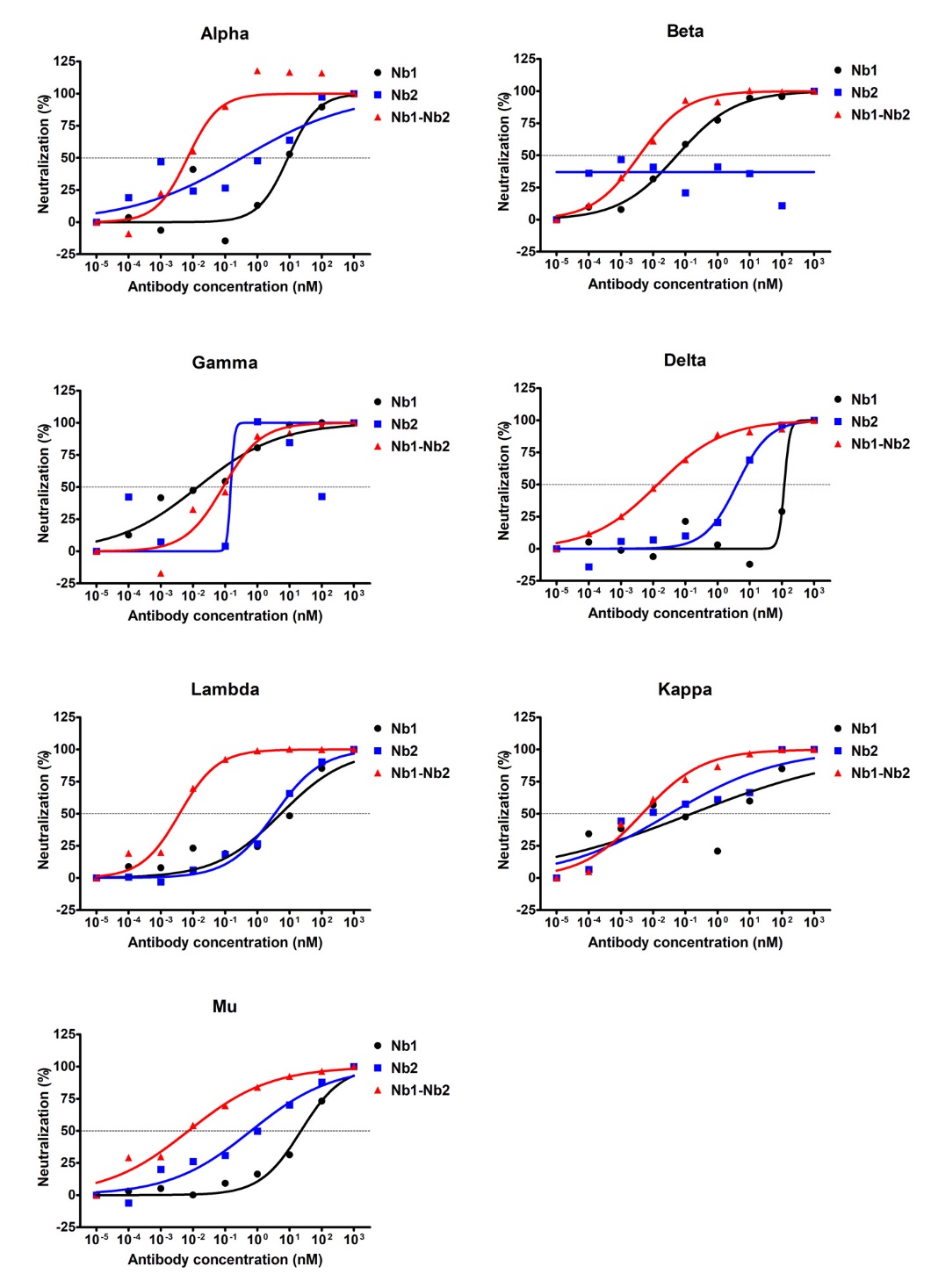


Figure. S1.

Neutralization of variants derived SARS-CoV-2 pseudoviruses by monomeric and bivalent Nbs.


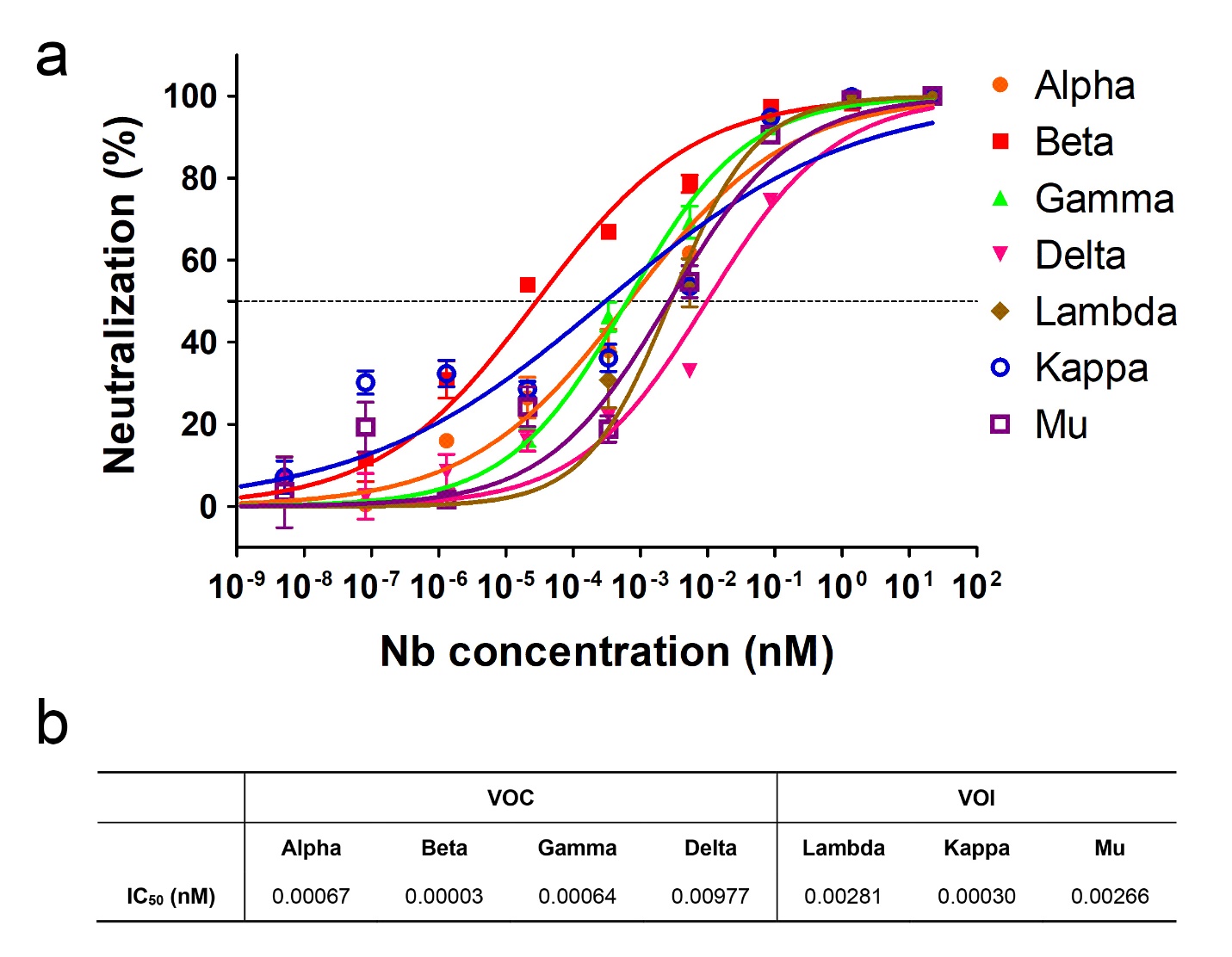


Figure. S2.

Neutralization of variants derived SARS-CoV-2 pseudoviruses by Nb1-Nb2-Fc.

**a** Neutralization curve of pseudoviruses by Nb1-Nb2-Fc. **b** Summary of the IC_50_ values.


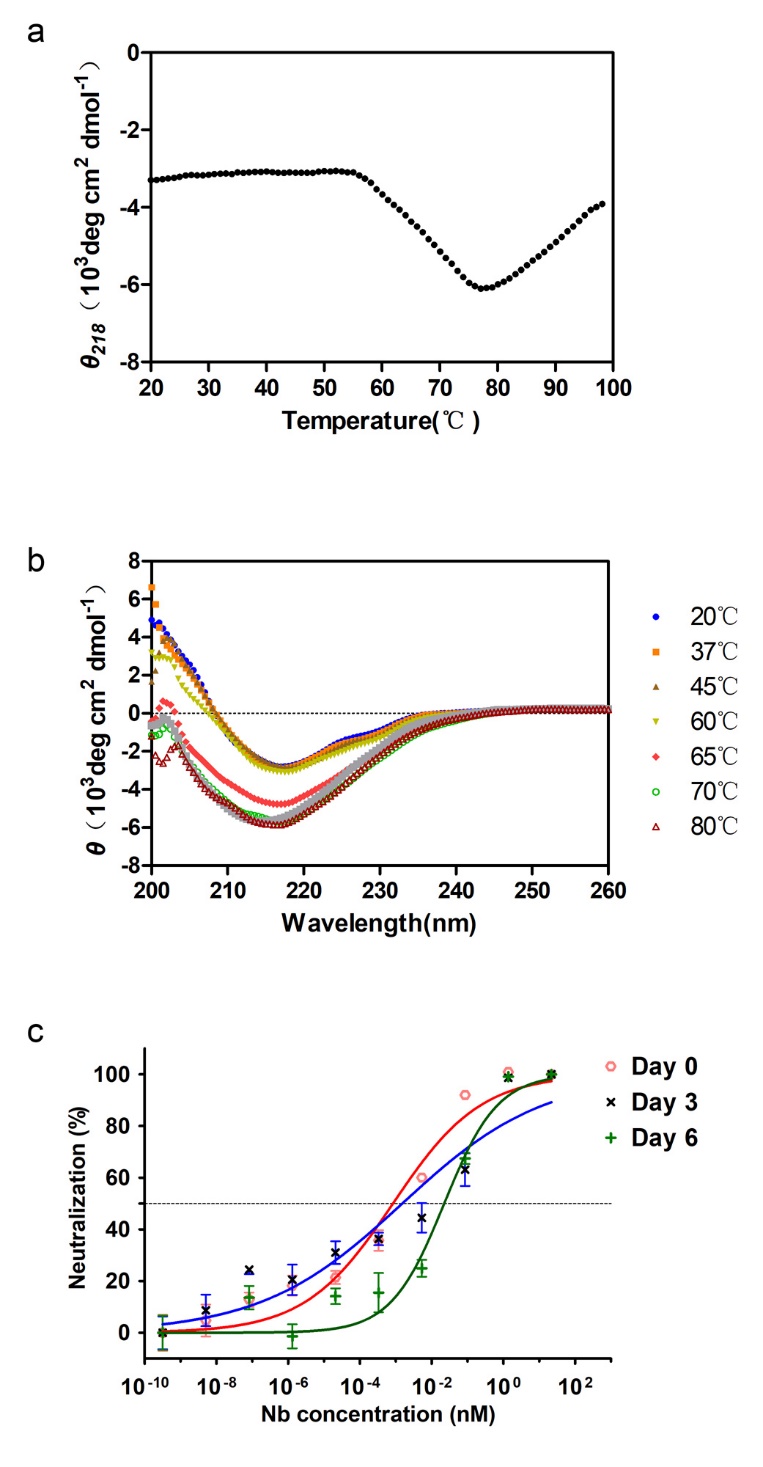


Figure. S3.

Thermal stability of Nb1-Nb2-Fc.

**a** Thermal melt of Nb1-Nb2-Fc was monitored by Circular dichroism (CD) at 218 nm at 2.7 µM protein concentration in PBS (pH 7.0). **b** CD spectra of Nb1-Nb2-Fc at a series of temperatures in PBS (pH 7.0). **c** Neutralization of SARS-CoV-2 pseudovirus by Nb1-Nb2-Fc that was stored in PBS at 37 ℃ for the indicated time courses without any protectants or protease inhibitors.


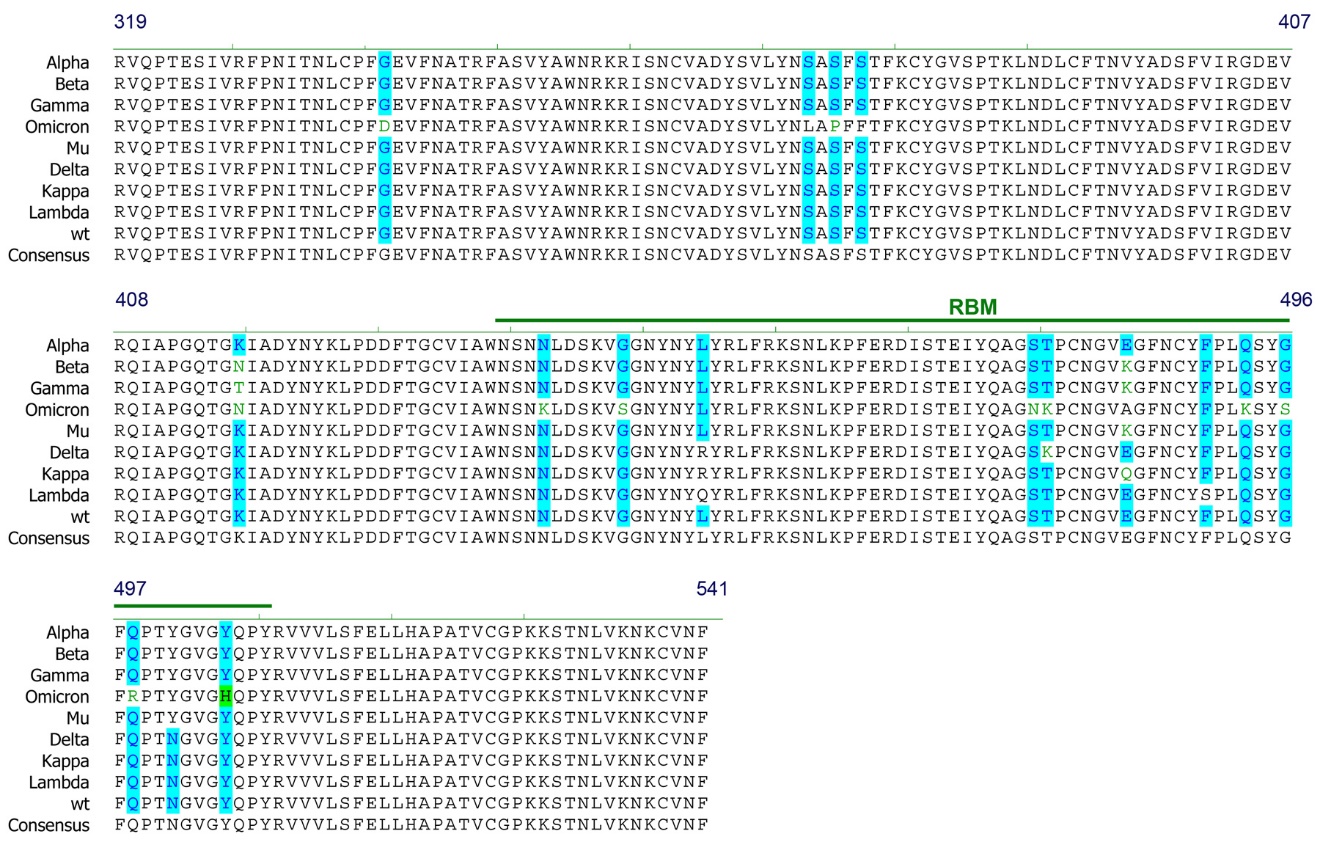


Figure. S4.

RBD amino acid sequence alignment from multiple VOC and VOI. Mutated points were colored. Consensus sequence was derived from the Wuhan isolate (wt).

Table S1.

Sequences of complementary determining regions

| **Nbs** | **CDR1** | **CDR2** | **CDR3** |
| --- | --- | --- | --- |
| **1** | grifevtwm | isrgggt | hqtvltsvewegyd |
| **2** | grilavfvm | ithggtt | dldynvyfhpywqlyd |
| **5** | gsifaaewm | inkggst | lilehqplgdeyyd |
| **7** | gfifhnidm | isfgsnt | yfyfqffeksehyd |
| **11** | gsilfvphm | inhgsnt | vvasldgelfesyd |
| **12** | gfifradrm | idfggdt | iyhhasiyehfayd |
| **13** | gfifpainm | itfgrit | rvveheqfnlld |
| **14** | grifvpvwm | insggrt | iyvkdlnddwanyd |
| **15** | grilwfnwm | isegrst | ittfftisewrgyd |
| **16** | gfifsttam | issgrit | rvhynefhnfrvyd |
| **17** | grifnvdym | iphgrst | iqqndltydyhtyd |
| **18** | gfifhrhpm | isngsit | iqqndltydyhtyd |
| **19** | gfilqfnpm | isfggit | nvqyagagkwyd |
| **20** | gfifrfrdm | indggit | kaqrythelwld |
| **23** | gfifhaidm | itnggit | qvfhllnttwyd |
| **24** | gfifhsipm | iagggit | qvyfhhrkhayd |
| **25** | gfifasagm | isnggit | qaqptkhshgld |
| **26** | gfifehinm | isfgfrt | rvqqgakpgprlshyd |

Table S2.

Primer design for pseudovirus mutagenesis

| **Oligonucleotides** | |
| --- | --- |
| **D614G(F)** | ctctgtgcaattcacgccttggtacagcacggc |
| **L18F(F)** | ctgggttctagtagtaaagttgacgcattggctac |
| **T19R(F)** | gagctgggttctagtattaaggttgacgcattgg |
| **L18F&T20N(F)** | gagggagctgggttctattagtaaagttgacgcattggctac |
| **P26S(F)** | agagttggtatatgctgaagggagctgggttctag |
| **69-70HV deletion(F)** | acttggtttcatgctatctctggaactaacggaacc |
| **G75V,T76I(F)** | gggttgtcaaagcgcttgattacgttagttccagacacat |
| **D80A(F)** | ggaggacggggttggcaaagcgcttggtt |
| **T95I(F) (F)** | gcgaattatgtttgacttttctatggaagcgaagtacacgccat |
| **D138Y(F)** | cccaggaagggataattgcagaactgaaattcacacac |
| **G142D(F)** | ttcttatggtagtacacatccaggaagggatcattgc |
| **144Y deletion(F)** | tcttgttattcttatggtacacacccaggaagggatc |
| **E154K(F)** | ctgtaaaccctaaattcggatttcatccagctcttgttattctta |
| **E156G(F)** | gaactgtaaaccctaaatccggattccatccagctct |
| **E156Gdel,G157/V158(F)** | gctggatggaatccggagtttacagttccgctaa |
| **157-158 deletion(F)** | gctggatggaatccgaagtttacagttccgctaa |
| **R190S(F)** | gatatttttgaacacgaactcgctcaagttcttgaagtttcct |
| **D215G(F)** | tccctggggcagatcgcgcaccaag |
| **L242-244 deletion(F)** | ctcgctttcaaacactgcatcgcagttacctcac |
| **R246I(F)** | ggggtgaggtaactgatatgcagggcgagcagt |
| **246-253 deletion(F)** | ccatcctgaactactatgcagggcgagcagtgt |
| **P337S(F)** | gttgaacacctcgccgaagctacacagatttgtaatgttg |
| **F338L(F)** | gttgaacacctcgcctaagggacacagatttgtaa |
| **V341I(F)** | gcgtgtggcgttgaatatctcgccgaagggaca |
| **F342L(F)** | gtgtggcgtttaacacctcgccgaaggga |
| **A344S(F)** | gaagcgaagcgtgtgctgttgaacacctcgcc |
| **R346K(F)** | atgcgtacactgaagcgaactttgtggcgttgaacacctcg |
| **A348S(F)** | ccatgcgtacactgaactgaagcgtgtggcgttg |
| **A352S(F)** | cgcttgcggttccatgagtacactgaagcgaag- |
| **N354D(F)** | gatatgcgcttgcggtcccatgcgtacactgaa |
| **S359N(F)** | atccgcgacgcagttatttatgcgcttgcggttcc |
| **V367F(F)** | gagttgtagaggaaagaataatccgcgacgcagtt |
| **N370S(F)** | ggagaaagaggcggagctgtagaggacagaataat |
| **A372S(F)** | cttgaaggtggagaaagagctggagttgtagaggacagaa |
| **A372T(F)** | gaaggtggagaaagaggtggagttgtagaggacag |
| **F377L(F)** | acactccgtagcactttaaggtggagaaagaggc |
| **K378N(F)** | tgacactccgtagcaattgaaggtggagaaagagg |
| **K378R(F)** | gtgacactccgtagcacctgaaggtggagaaagag |
| **P384L(F)** | gatcgttcagcttagtcagtgacactccgtagcac |
| **T385A(F)** | agatcgttcagcttagccggtgacactccgtag |
| **T393P(F)** | gcgtagacgttgggaaagcagagatcgttcagctt |
| **V395I(F)** | gaaggagtccgcgtagatgttggtaaagcagagat |
| **E406Q(F)** | ggggctatttgtctcacctgatcacctcttatcacgaagga |
| **R408I(F)** | tggggctatttgtatcacttcatcacctcttatcacga |
| **Q409E(F)** | gtctgacctggggctatctctctcacttcatcacctc |
| **Q414E(F)** | cgatcttaccagtctcacctggggctatttgtc |
| **Q414R(F)** | tgcgatcttaccagtccgacctggggctatttg |
| **K417T(F)** | agttgtaatctgcgatcgtaccagtctgacctggg |
| **K417N(F)** | gtaatctgcgatattaccagtctgacctggggct |
| **A435S(F)** | ggttattagagttccacgagataacgcaaccagtgaaa |
| **W436R(F)** | gaggttattagagttcctcgcgataacgcaaccag |
| **N439K(F)** | gaccttagaatcgaggttcttagagttccacgcgataac |
| **N440K(F)** | ccttagaatcgagcttattagagttccacgcgataacg |
| **K444R(F)** | gttaccaccgaccctagaatcgaggttattagagttcc |
| **L452Q(F)** | ttcctaaacaagcggtactggtaattgtagttaccacc |
| **L452R(F)** | ttcctaaacaagcggtaccggtaattgtagttaccacc |
| **Y453F(F)** | ctaaacaagcggaacaggtaattgtagttaccaccg |
| **K458R(F)** | aaggcttcaggtttgacctcctaaacaagcggtac |
| **E471Q(F)** | ttgaacccgcttgatagatctgggttgaaatatccctctcg |
| **I472V(F)** | acccgcttgatagacttcggttgaaatatccctctc |
| **G476S(F)** | gttacacggtgttgaactcgcttgatagatttcgg |
| **S477I(F)** | cacaccgttacacggtgttatacccgcttgatagatttcg |
| **S477R(F)** | cacaccgttacacggtgttctacccgcttgatagatttcg |
| **T478K(F)** | ccgttacacggttttgaacccgcttgatagatttc |
| **G482S(F)** | agttaaatccttccacactgttacacggtgttgaacc |
| **V483A(F)** | agcagttaaatccttccgcaccgttacacggtgtt |
| **V483I(F)** | gaagtagcagttaaatccttctataccgttacacggtgttgaacc |
| **E484Y(F)** | ggaagtagcagttaaatccatacacaccgttacacggtgtt |
| **E484Q(F)** | ggaagtagcagttaaatccctgcacaccgttacacggtgtt |
| **E484K(F)** | gtagcagttaaatcctttcacaccgttacacggtg |
| **G485S(F)** | ggggaagtagcagttaaagctttccacaccgttacacgg |
| **F490S(F)** | agactgcaggggaaagtagcagttaaatccttccac |
| **P497S(F)** | caccgttacacgatgttgaacccgcttgatagat |
| **S494P(F)** | gctggaatccgtaaggctgcagggggaagta |
| **N501Y(F)** | ggtaacccacgccataggttggctggaatcc |
| **V503F(F)** | cgataaggttggtaaccgaagccattggttggctgga |
| **Y508H(F)** | gaaccaccacgcgatgaggttggtaacccac |
| **A520S(F)** | gcataccgtggcgggactgtgcaacagttcg |
| **P521R(F)** | gcataccgtggcgcgagcgtgcaacag |
| **P521S(F)** | cataccgtggcgctagcgtgcaacagttcgaaactca |
| **A522S(F)** | gaccgcataccgtgctgggagcgtgcaaca |
| **A522V(F)** | ccgcataccgtgacgggagcgtgca |
| **A570D(F)** | cgcgtcggttgtgtcatcaatgtctcttccgaa |
| **H665Y(F)** | gtaggagttattcacatattccgctccgatcaagca |
| **P681H(F)** | ctgcgggctctcctgtgtgagttagtttgtg |
| **P681R(F)** | ctgcgggctctcctgcgtgagttagtttgtg |
| **T716I(F)** | cacgctaattgtgaagttgattgggatggcgattgaatt |
| **T859N(F)** | tcagaagtgggggcaagacattcaggccattaaacttctg |
| **D950N(F)** | attctggttcaccacgttctgcaacttaccaaggg |
| **T1027Y(F)** | gcattcggacatcttgattgcagccagattggc |
| **E1072K, K1073R(F)** | ggcgcagtggtaaaattccttttctgtgctggaacgtaggt |
| **H1101D(F)** | ctgtgtgacgaaccaatcagttccgttagacacga |
| **D1118H(F)** | cggacacaaatgtattgtgggtggtaatgatctgagg |
| **V1176F(F)** | gtcaatctctttttgaatattgacgaaggacgcattgataccactaatgt |

Table S3.

Variants of SARS-CoV-2 spike amino acid changes.

| **Variant** | **Amino acid changes** |
| --- | --- |
| **Alpha** | 69-70HV del, 144Ydel，N501Y,,P681H，T716I, S982A, D1118H, D614G, A570D |
| **Beta** | L18F, D80A, D215G, LAL242-244 deletion, R246I, K417N, E484K, N501Y, D614G, A701V |
| **Gamma** | L18F，T20N，P26S，D138Y，R190S，K417T,E484Y, N501Y,D614G, H655Y，T1027I，V1176F |
| **Delta** | T19R, G142D, 157-158del, E156G, L452R, T478K, D614G, P681R, D950N |
| **kappa** | T95I, G142D, E154K, L452R, E484Q, D614G, P681R, Q1071H |
| **Lambda** | G75V, T76I,246-252DEL, L452Q, F490S, D614G, T859N, M95I, Y144S, Y145N, R346K, E484K, N501Y, D614G, P681H, D950N |
| **Omicron** | A67V, Δ69-70, T95I, G142D, Δ143-145, N211I, Δ212, ins215EPE, G339D, S371L, S373P, S375F, K417N, N440K, G446S, S477N, T478K, E484A, Q493R, G496S, Q498R, N501Y, Y505H, T547K, D614G, H655Y, N679K, P681H, N764K, D796Y, N856K, Q954H, N969K, L981F |
