# Supplementary material for: An ultrapotent RBD-targeted biparatopic nanobody neutralizes broad SARS-CoV-2 variants
Source: Signal Transduct Target Ther. 2022 Feb 9;7:44. doi: 10.1038/s41392-022-00912-4 (PMC8828845; doi:10.1038/s41392-022-00912-4)
Supplement: Supplementary file 2 — Source Data [file 41392_2022_912_MOESM2_ESM.zip › Fig 3a/IC50 against mutation pseudoviruses.docx]

|  | **IC50 (nM)** | | |  | **IC50 (nM)** | | |  | **IC50 (nM)** | | |
| --- | --- | --- | --- | --- | --- | --- | --- | --- | --- | --- | --- |
|  | **Nb1-3** | **Nb2-1** | **Bi-1-2** |  | **Nb1-3** | **Nb2-1** | **Bi-1-2** |  | **Nb1-3** | **Nb2-1** | **Bi-1-2** |
| **D614G wt** | 0.0666 | 0.1323 | 0.00635 | **Q414E** | 0.01183 | 0.04259 | 0.000732 | **F490S** | 0.05018 | 0.1667 | 0.001619 |
| **F338L** | 0.1554 | 0.5516 | 0.001459 | **K417T** | 0.1724 | 2.347 | 0.002786 | **S494P** | 4.14 | 0.3003 | 0.03089 |
| **V341I** | 0.009006 | 0.1366 | 0.003611 | **K417N** | 0.9749 | 15.78 | 0.000931 | **N501Y** | 3.019 | 0.0796 | 0.005896 |
| **F342L** | 0.35 | 0.1608 | 0.002492 | **A435S** | 0.1825 | 0.289 | 0.000238 | **V503F** | 0.08093 | 0.2602 | 0.000527 |
| **A344ST** | 0.07888 | 0.8067 | 0.003694 | **N439K** | 0.8943 | N | 0.003824 | **Y508H** | 1.166 | 0.4534 | 0.006157 |
| **A348S** | 67.72 | 0.01264 | 0.001999 | **N440K** | 0.01729 | 0.2337 | 0.007845 | **A520S** | 1.566 | 0.062 | 0.007126 |
| **A352S** | 1.051 | 0.3646 | 0.01124 | **K444R** | 0.5603 | 0.4327 | 0.003002 | **P521S** | 1.32 | 0.6323 | 0.005776 |
| **N354D** | N | 0.3744 | 0.03121 | **L452L** | 3.189 | 0.09071 | 0.002374 | **P521R** | 0.1263 | 0.101 | 0.000555 |
| **S359N** | 0.06718 | 0.4674 | 0.000565 | **K458R** | 0.01599 | 0.01539 | 0.007204 | **A522V** | 1.601 | 0.7029 | 0.002943 |
| **V367F** | 0.0364 | 0.1177 | 0.001539 | **E471Q** | 10.05 | 0.09232 | 0.00346 | **A522S** | 0.1186 | 0.002714 | 0.002312 |
| **N370S** | 0.3745 | 0.002832 | 0.01146 | **I472V** | 0.198 | 0.08409 | 0.000553 | **P618H** | 5.798 | 0.6318 | 0.0083 |
| **F377L** | 0.05122 | 0.1511 | 0.00015 | **G476S** | 0.05512 | 0.00565 | 0.007889 | **P618R** | 0.414 | 0.05206 | 0.004949 |
| **K378R** | 0.1371 | 1.795 | 0.000237 | **S477N** | 0.05025 | 0.05826 | 0.003793 | **P681H** | 1.474 | 0.0141 | 0.007728 |
| **K378N** | 0.938 | 0.5094 | 0.001091 | **S477R** | 0.4218 | 0.1569 | 0.004516 | **T716I** | 0.01854 | 0.01897 | 0.003089 |
| **P384L** | 0.1534 | 0.1328 | 0.000623 | **T478I** | 0.02154 | 0.6199 | 0.000332 | **S982A** | 0.7595 | 1.05 | 0.002567 |
| **T385A** | 0.3215 | 0.01048 | 0.000672 | **P479S** | 2.345 | 0.1186 | 0.001188 | **D1118H** | 1.919 | 0.2633 | 0.001811 |
| **T393P** | N | 0.02477 | 0.001285 | **G482S** | 0.05337 | 0.02668 | 0.000589 | **L452R/P681R** | 6.827 | 1.15 | 0.001773 |
| **V395I** | 0.09371 | 0.9681 | 0.001914 | **V483A** | 0.4289 | 1.767 | 0.002712 | **N501Y/D1118H** | 1.384 | 0.5301 | 0.001989 |
| **E406Q** | 2.191 | 9.164 | 0.01657 | **V483I** | 0.01564 | 0.2966 | 0.000419 | **P681R/L452R/E484Q** | 9.062 | 3.438 | 0.01575 |
| **R408I** | 0.008322 | 0.1279 | 0.000427 | **E484K** | 14.3 | N | 0.03511 | **69H70V del** | 0.03105 | 0.4216 | 0.00116 |
| **Q409E** | 1.417 | 1.798 | 0.007547 | **E484Q** | 19.82 | 19.32 | 0.04036 | **144Y del** | 0.0956 | 0.08331 | 0.003605 |
| **Q414R** | 1.052 | 0.3085 | 0.001102 | **G485S** | 0.1167 | 0.01903 | 0.000805 |  |  |  |  |
