# Supplementary figures and images for: An ultrapotent RBD-targeted biparatopic nanobody neutralizes broad SARS-CoV-2 variants
Source: Signal Transduct Target Ther. 2022 Feb 9;7:44. doi: 10.1038/s41392-022-00912-4 (PMC8828845; doi:10.1038/s41392-022-00912-4)

# Caco-2-N cell (without trVLP infection)

#1

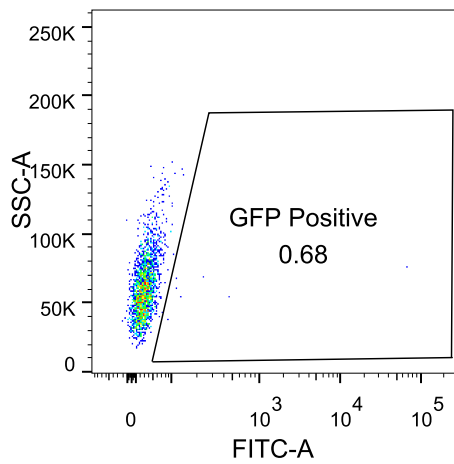

#2

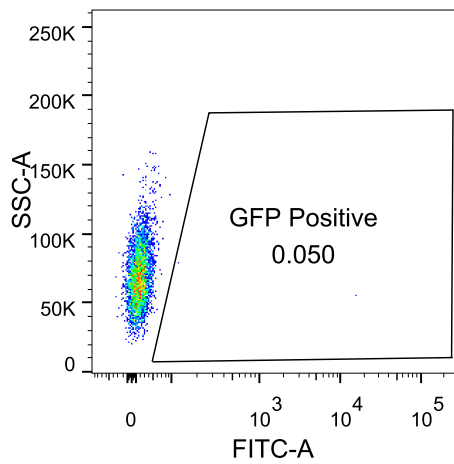

#3

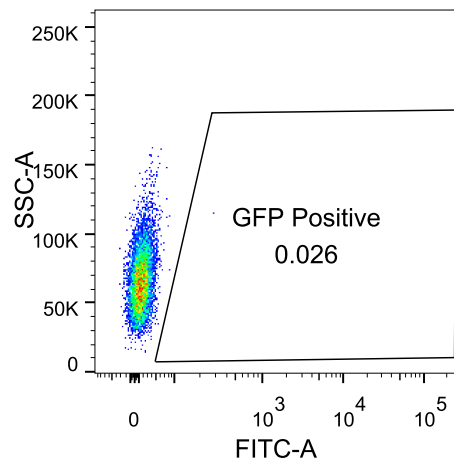

WT

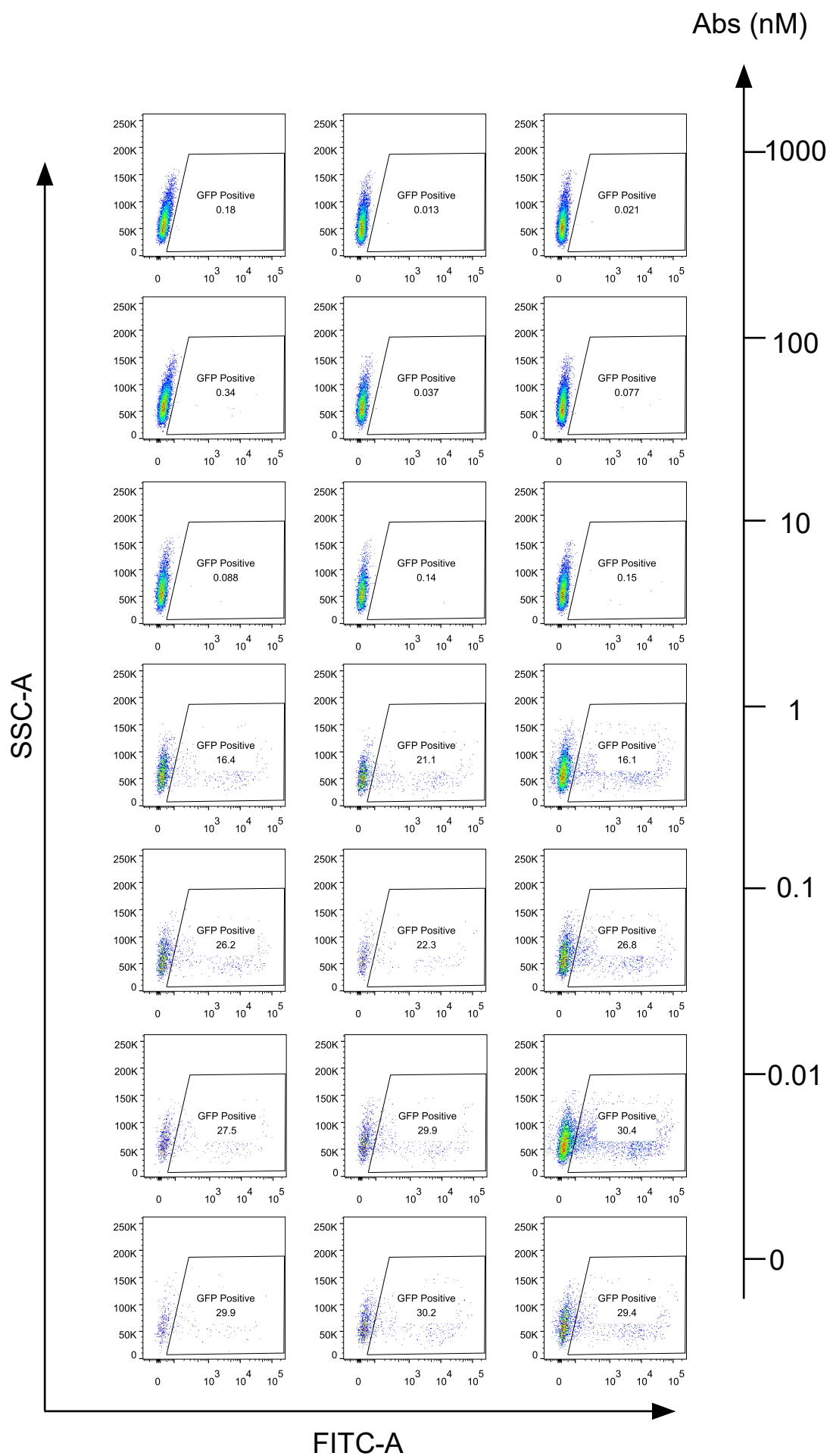

Alpha

Abs (nM)

SSC-A

FITC-A

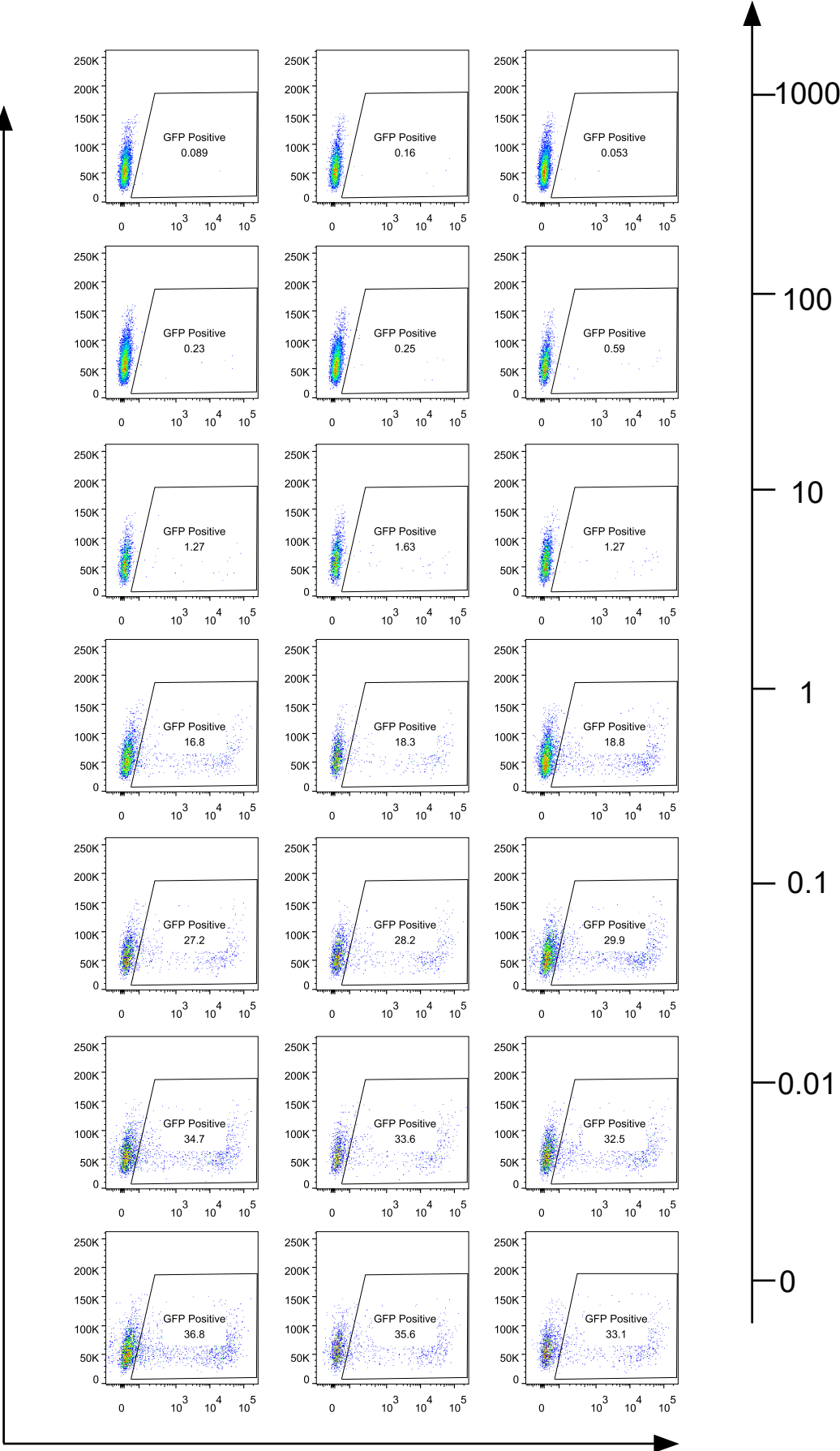

Beta

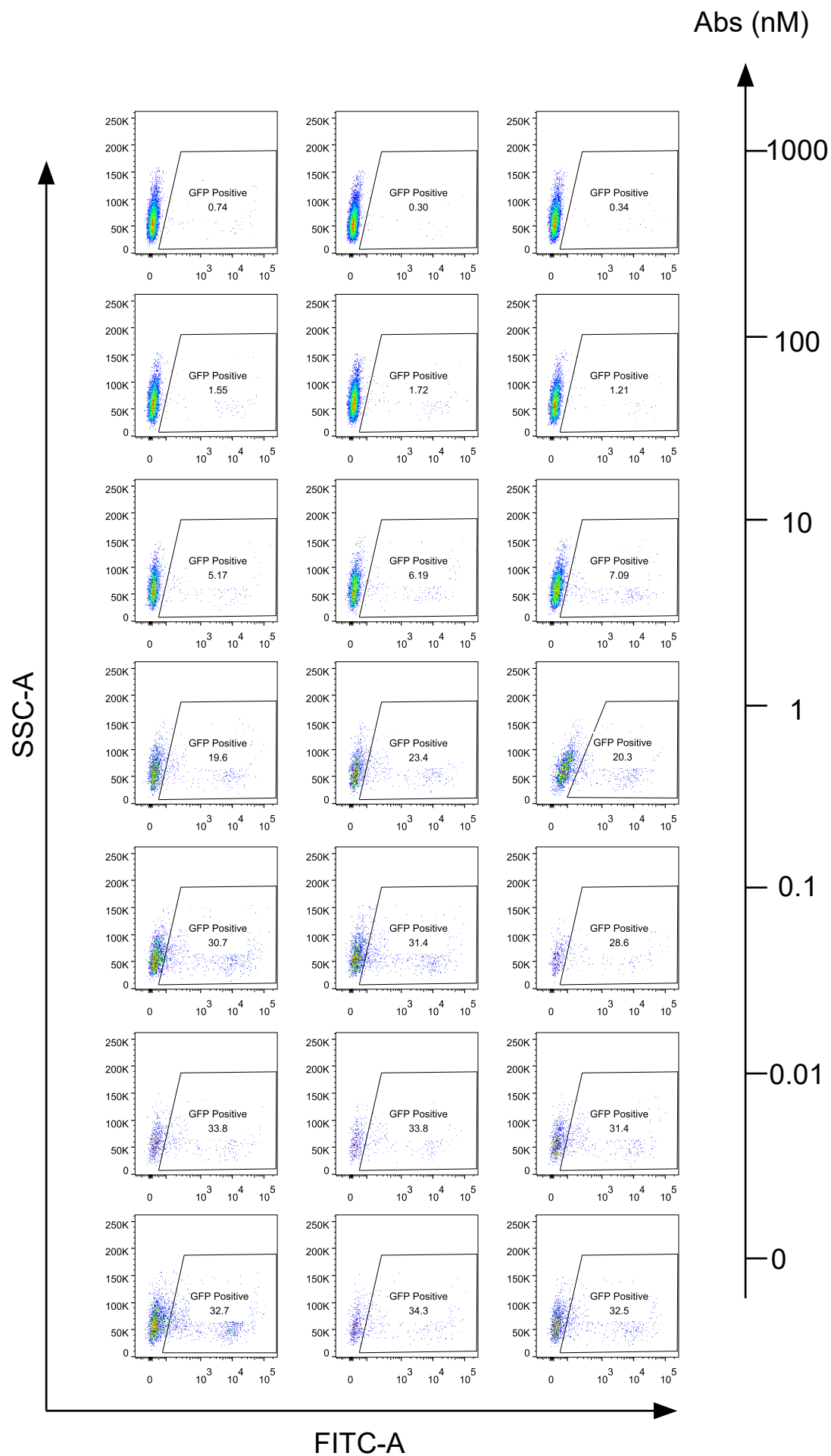

# Gamma

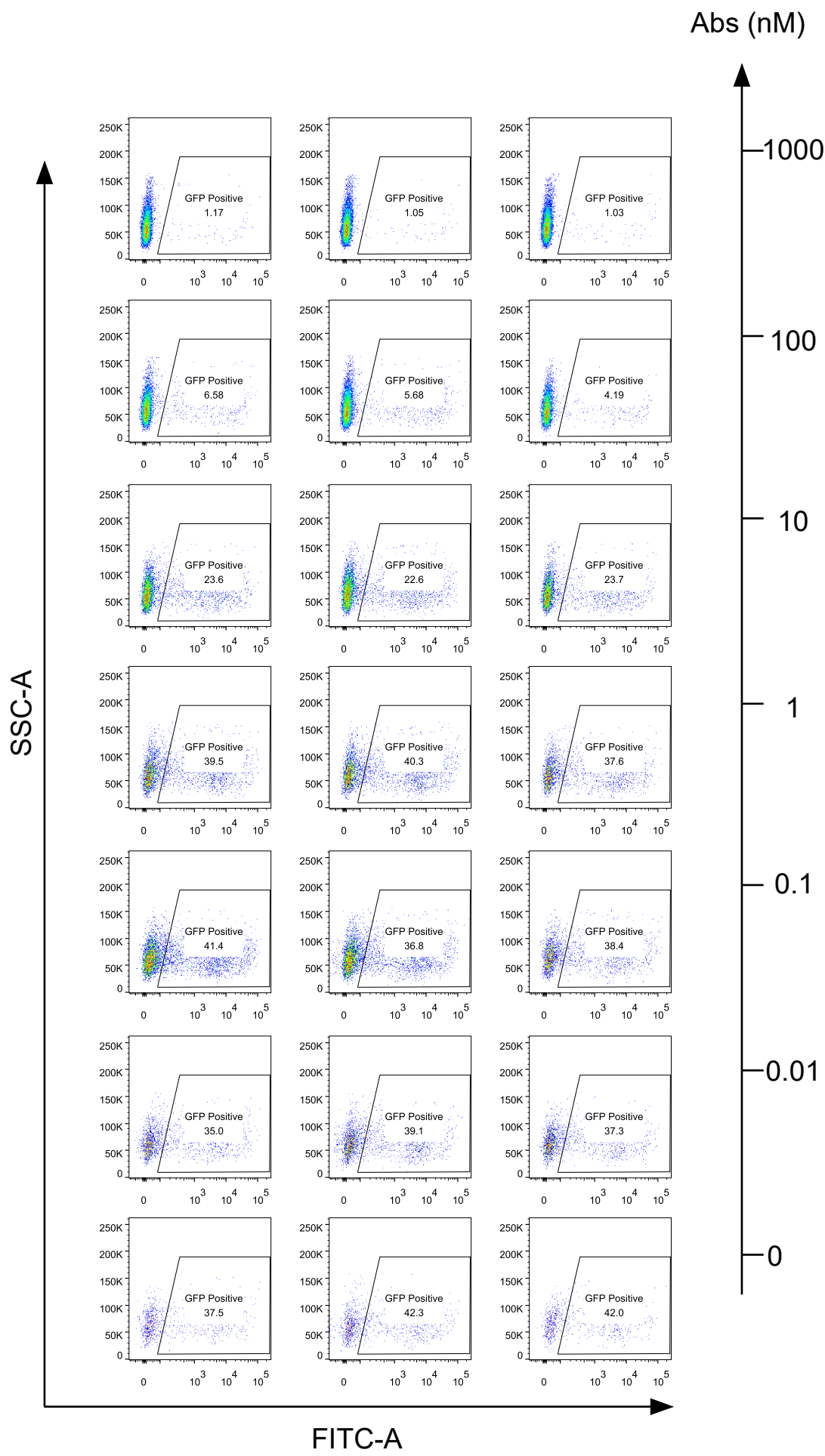

Delta

Abs (nM)

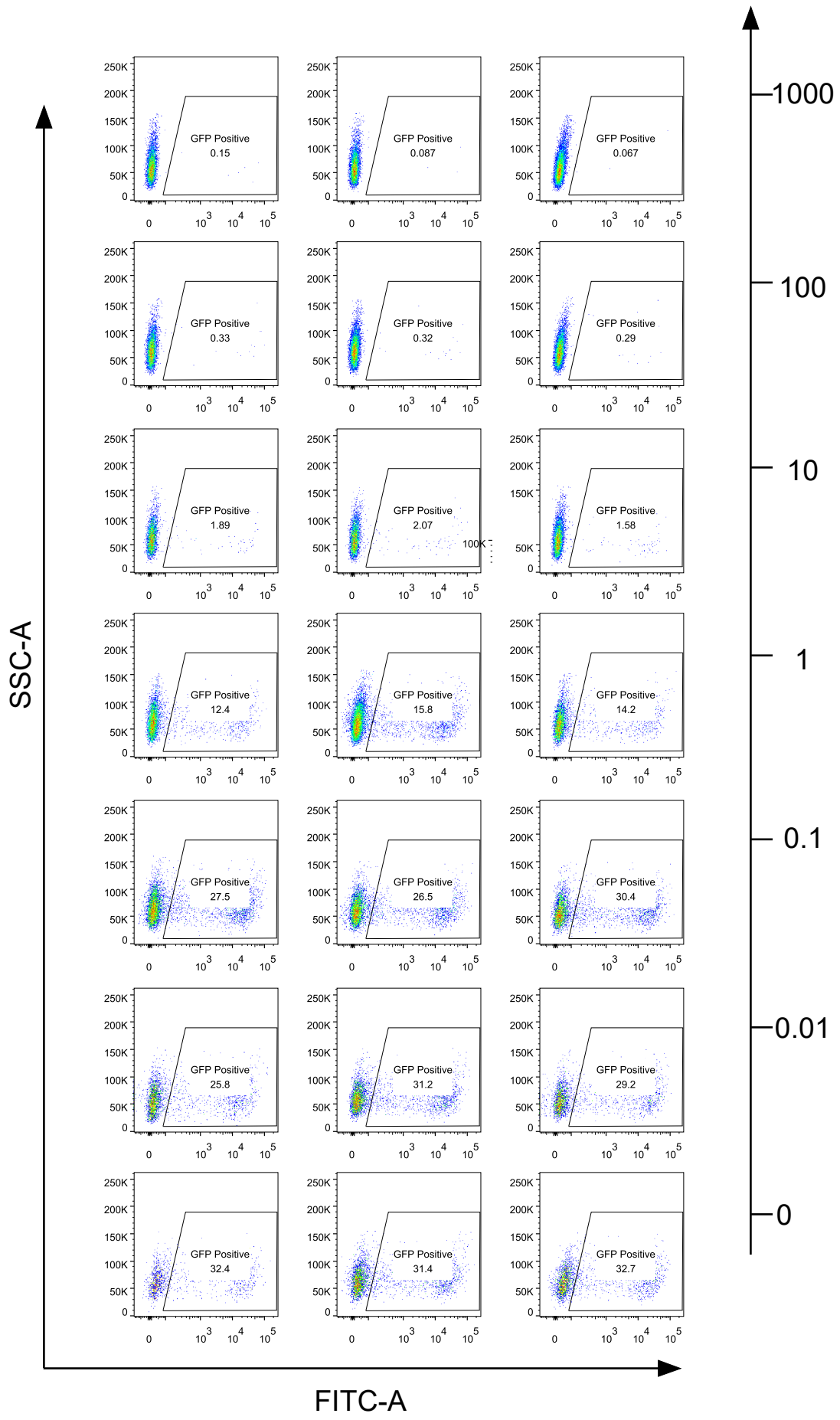

Supplement: Supplementary file 2 — Source Data [file 41392_2022_912_MOESM2_ESM.zip › Fig 4b/FACS data.pdf]

NC

#1

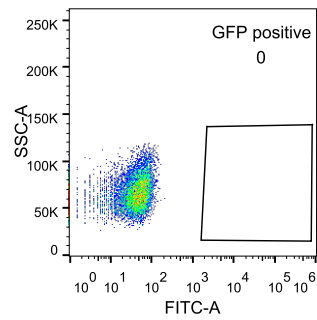

#2

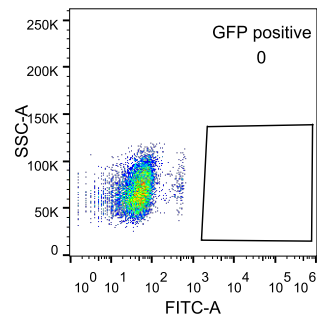

#3

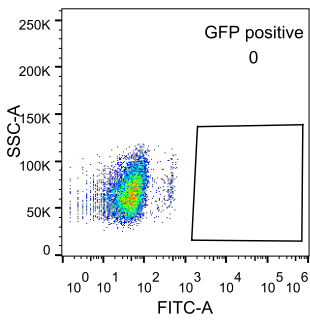

WT

SSC-A

FITC-A

Abs (nM)

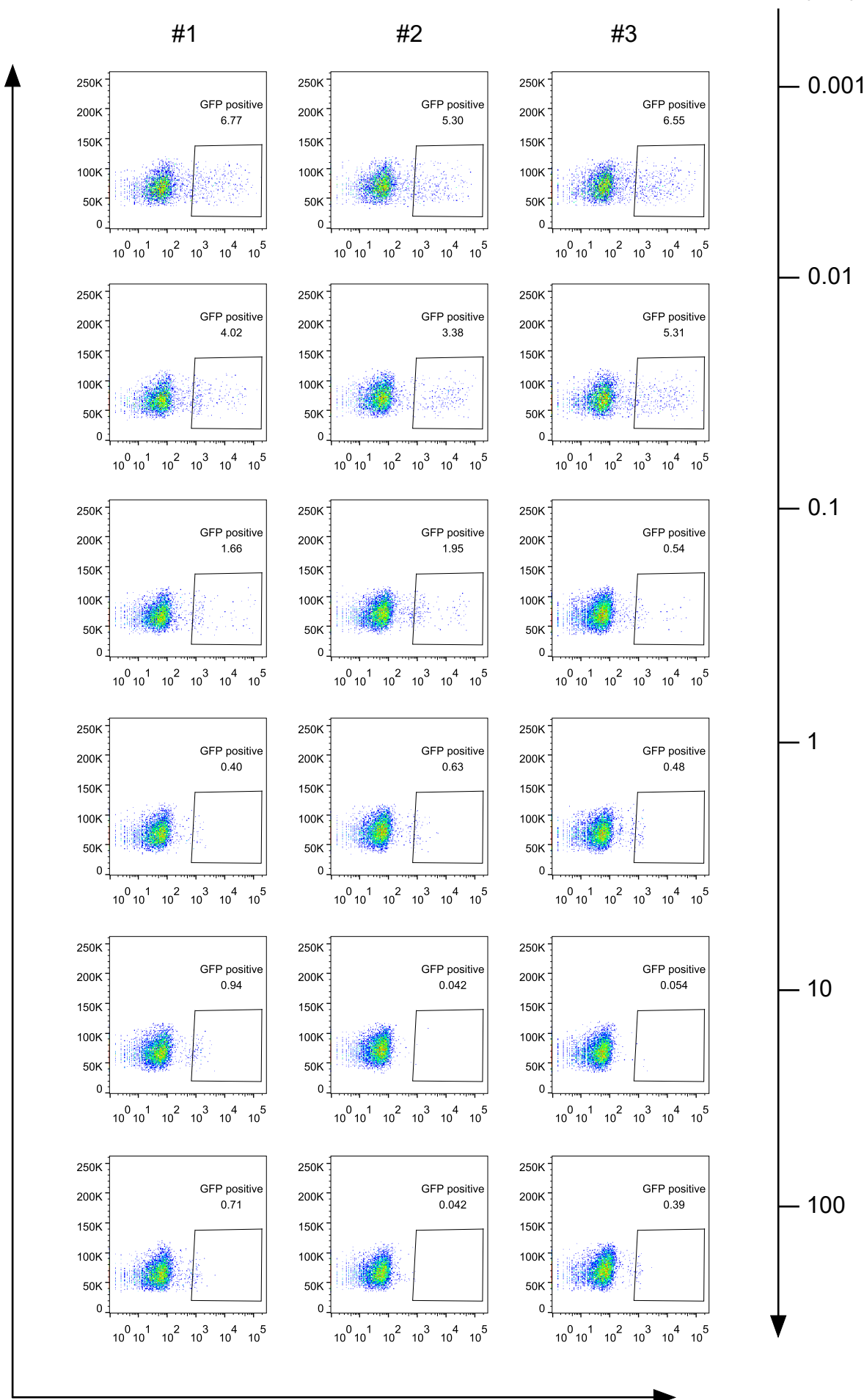

# Alpha

Abs (nM)

## #1

#2

### #3

SSC-A

FITC-A

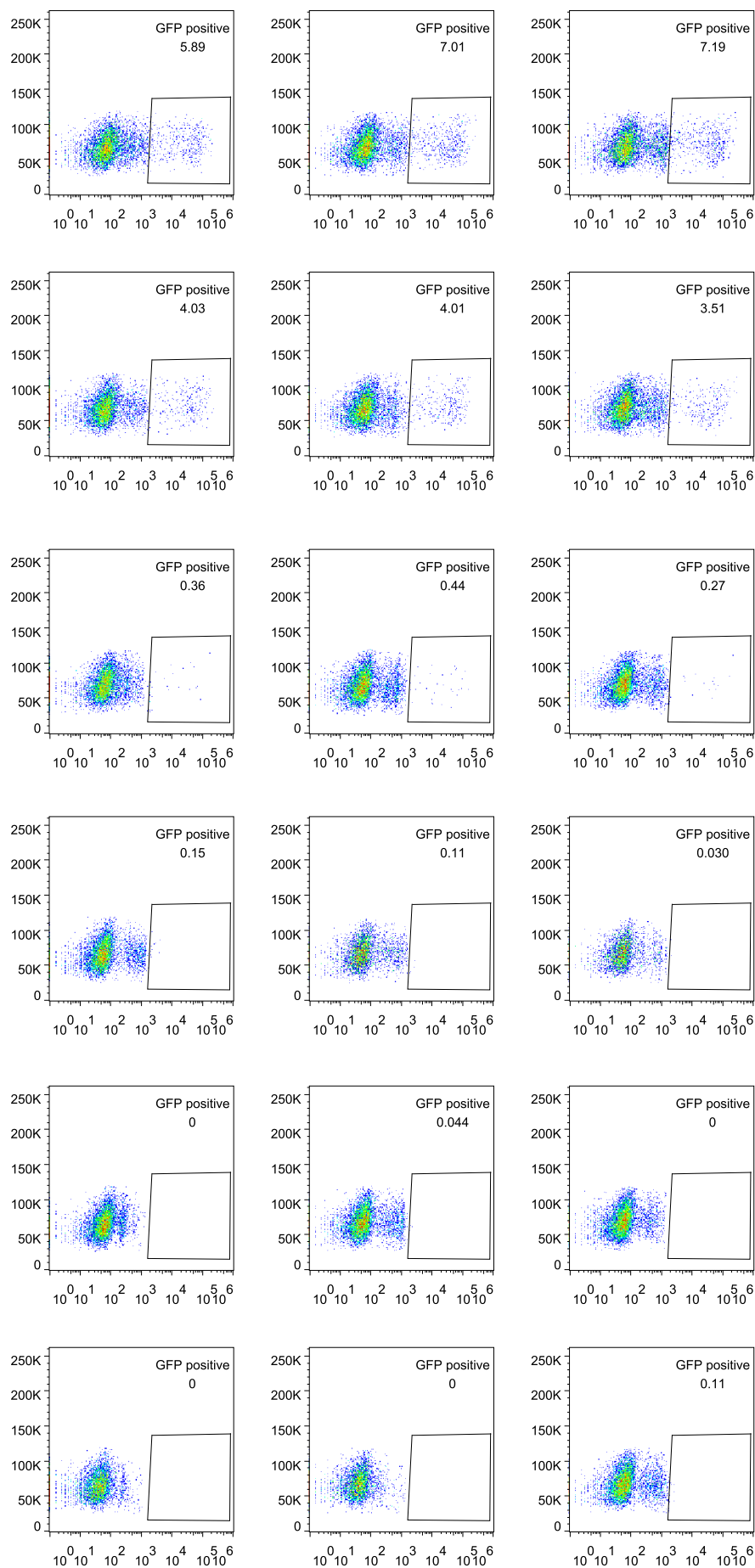

Beta

SSC-A

#1

#2

#3

Abs (nM)

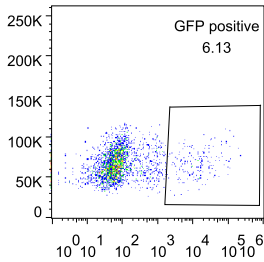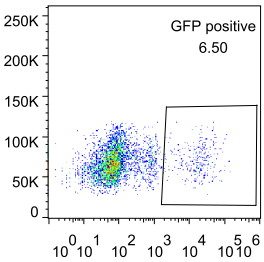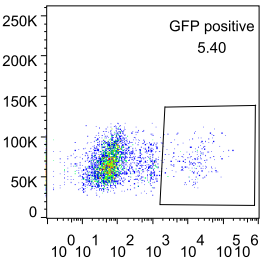

0.001

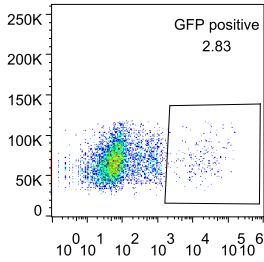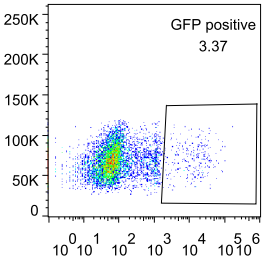

0.01

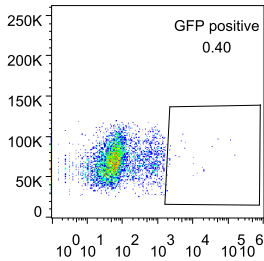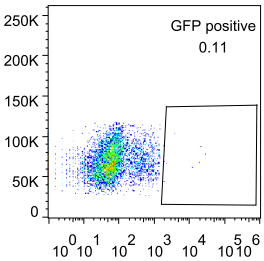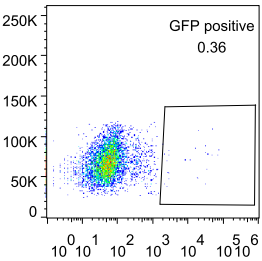

0.1

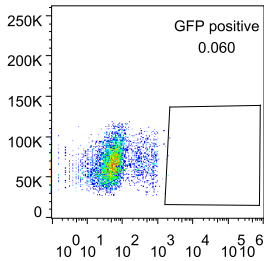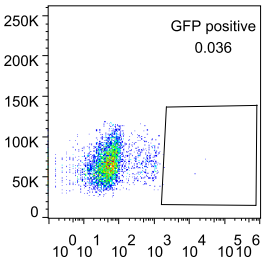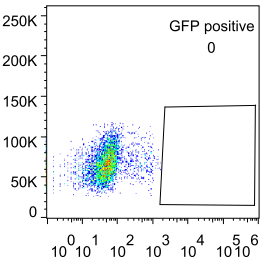

1

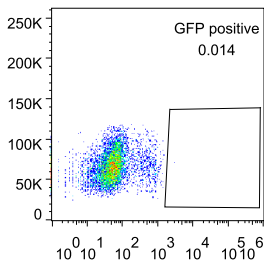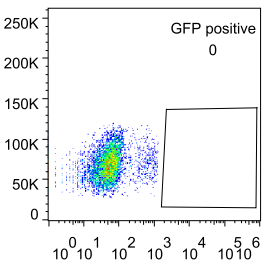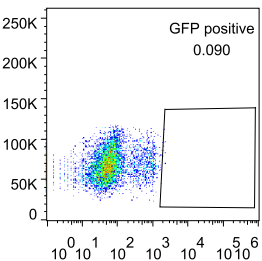

10

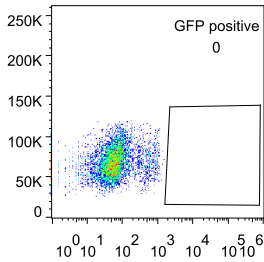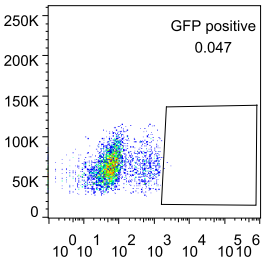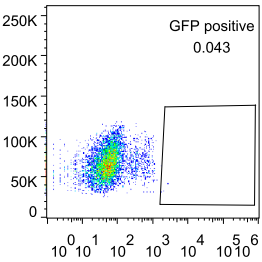

100

FITC-A

Gamma

SSC-A

FITC-A

Abs (nM)

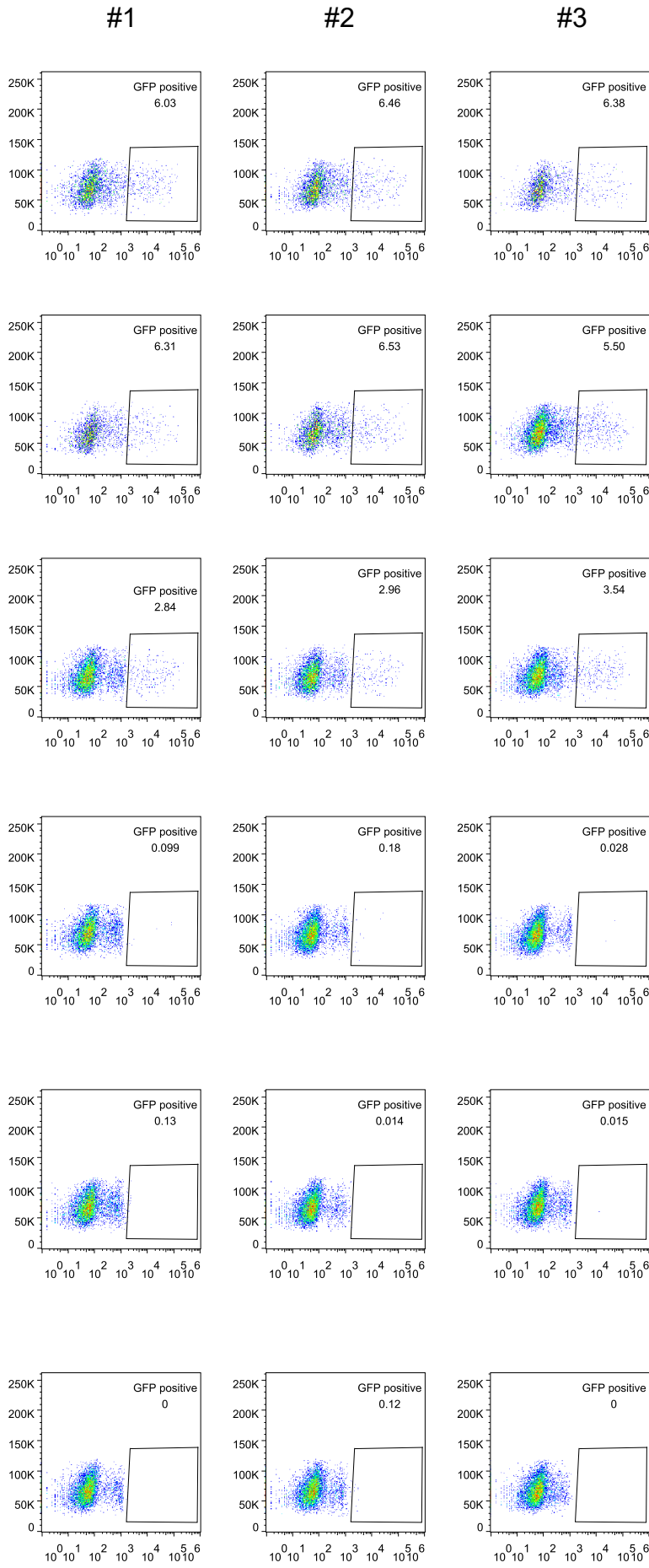

Delta

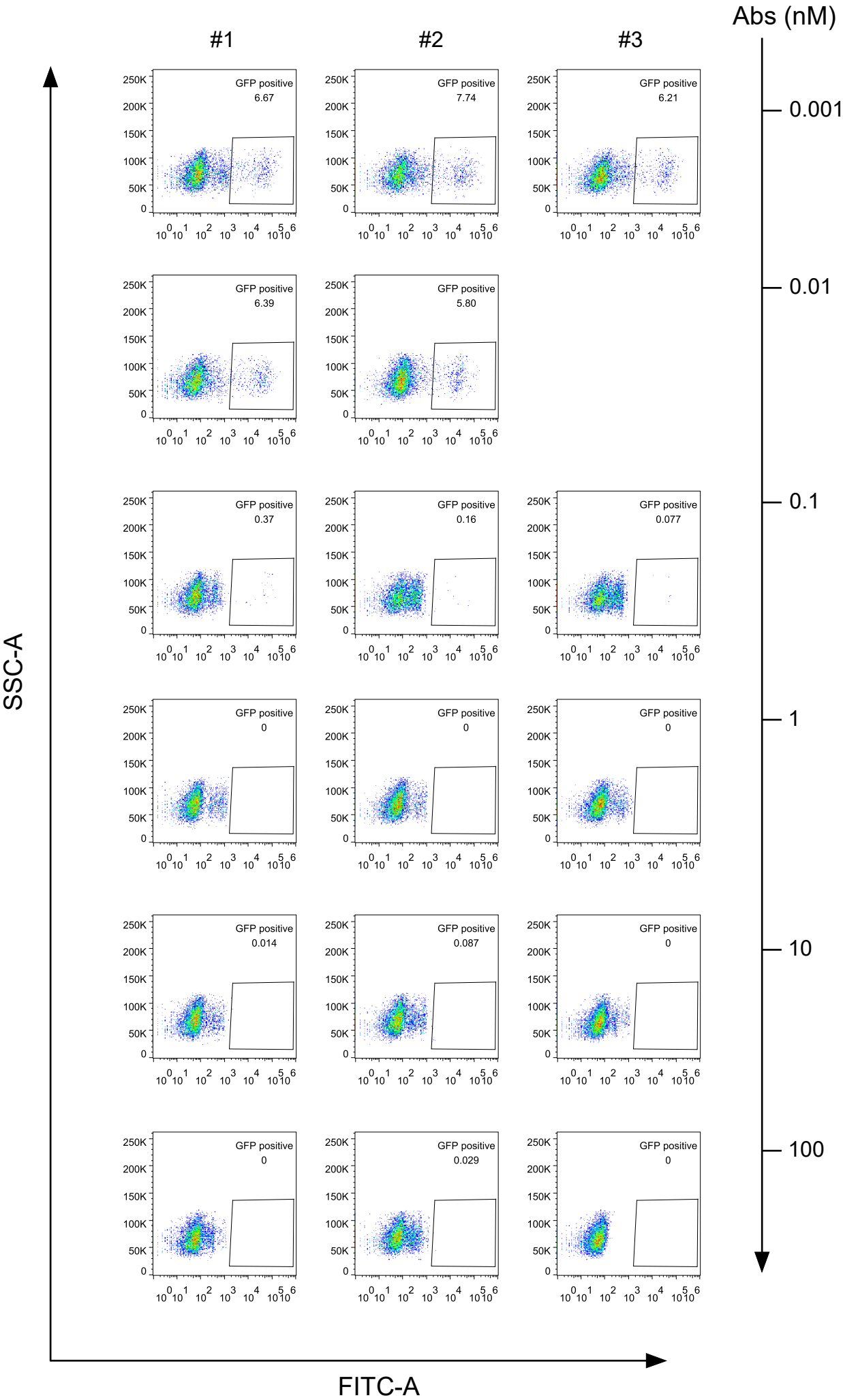

Supplement: Supplementary file 2 — Source Data [file 41392_2022_912_MOESM2_ESM.zip › Fig 5c/FACS Nb1-Nb2-Fc raw data.pdf]
